# Why Does Your Uterus Become Malignant? The Impact of the Microbiome on Endometrial Carcinogenesis

**DOI:** 10.3390/life13122269

**Published:** 2023-11-28

**Authors:** Katarzyna Morańska, Monika Englert-Golon, Magdalena Durda-Masny, Stefan Sajdak, Marlena Grabowska, Anita Szwed

**Affiliations:** 1 Institute of Human Biology and Evolution, Faculty of Biology, Adam Mickiewicz University, Uniwersytetu Poznanskiego 6, 61-614 Poznan, Polandaniszwed@amu.edu.pl (A.S.); 2 Department of Gynaecology Obstetrics and Gynaecological Oncology, Division of Gynecological Surgery, Poznan University of Medical Sciences, 60-535 Poznan, Poland

**Keywords:** endometrium, cancer, microbiome

## Abstract

The aim of this review was to describe the uterine microbiome composition that has been analyzed so far and describe potential pathways in the carcinogenesis of the endometrium. The microbiome in the uterine environment is involved in apoptosis and proliferation during the menstruation cycle, pregnancy maintenance, and immune system support. However, bacteria in the uterus could stimulate inflammation, which when chronic results in malignancy. An altered gut microbiota initiates an inflammatory response through microorganism-associated molecular patterns, which leads to intensified steroidogenesis in the ovaries and cancers. Moreover, intestinal bacteria secreting the enzyme β-glucuronidase may increase the level of circulating estrogen and, as a result, be influential in gynecological cancers. Both the uterine and the gut microbiota play a pivotal role in immune modulation, which is why there is a demand for further investigation from both the diagnostic and the therapeutic perspectives.

## 1. Introduction

Endometrial cancer (EC) is the sixth most common cancer among women in the world. According to global statistics, EC affects over 400,000 patients annually, which makes it the second most common gynecological cancer after cervical cancer [1]. In 2020, 417,367 women were diagnosed with EC worldwide, which accounts for 4.5% of all malignant neoplasms in women. North America has the highest incidence, followed by Eastern Europe (21.1 and 20.2 per 100,000 women, respectively). Epidemiologically, in 2020, most of the cases of EC were observed in Poland (9869 cases; 26.2 ASR (age standardized rate)), followed by Lithuania (803; 25.4 ASR), Samoa (20; 24.7 ASR), Belarus (2169; 23.6), Jamaica (421; 22.3), Ukraine (9705; 22.1), North Macedonia (369; 21.8), Bahamas (55; 21.8), the U.S. (61,738; 21.4), and Trinidad and Tobago (225; 20.5) [1,2]. There were 417,367 cases worldwide, collectively resulting in an 8.7 ASR. The American Institute for Cancer Research described EC as a disease of high-income countries, emphasizing North America and Central and Eastern Europe [2]. Researchers propound that the increasing incident rates are boosted in countries with societies undergoing the transition from low- to high-income economies. Accordingly, in the USA, rates are higher in White women than among other ethnic groups, although mortality rates are higher in Black women [3,4]. Commonly, EC risk increases with age, and EC is diagnosed mainly in postmenopausal women aged 55–65. The overall 5-year survival rate is relatively high. For all EC cases, it is about 69% [5].

The uterine microbiome’s composition varies in different pathologies. The latest data obtained by researchers from Mayo Clinic, Rochester, MN, USA, reported several bacterial taxa correlated with EC. The available studies’ results differ, and uterine bacteria demand further investigation. Undoubtedly, the microbiome plays a role in pathogenesis and could be useful in disease detection in the upper reproductive tract. Favorable species occupy the endometrial area and prevent pathogenic microorganisms from attaching, providing anti-infection abilities [6,7]. On the other hand, microbial ligands can be bound to host receptors and take part in an immune response by triggering the production of chemokines, inflammatory cytokines, and antibacterial substances [6,8]. The association between the microbiome and EC development is derived from local microbial imbalance and immune inducement. The microbiota, which stimulates inflammation, may induce immunopathological changes, which lead to tumors eventually [9]. If inflammation occurs, malignant transformation is standard, but pathways specific to the endometrium require further investigation. Finally, analyses of the relationship between the microbiome and the immune system, including gene expression changes, are needed in this field. The influence of the gut–vaginal microbiome axis on the endometrium [10,11] has been presented, with different microbial compositions within healthy individuals and in patients with cancer. The intestinal microbiota plays a key role in the level of circulating estrogen. Disturbed composition of the intestinal microbiota and lower diversity adversely affect the level of circulating estrogen, contributing to the development of obesity, metabolic syndromes, and cancer [11].

Both the intestinal and the gynecological microbiomes could be potentially influential in carcinogenesis through estrogen metabolism regulation, inflammation incitement locally, or accompanying disease conduction. The uterine microbiome is under further investigation, providing more data about the bacterial composition and influential pathways. The combination of those may lead to the development of EC prognostic and therapeutic agents. The aim of this review is to describe the uterine microbiome composition that has been analyzed so far and analyze potential pathways in the carcinogenesis of the endometrium.

## 2. Review Methodology

**Figure 1:**
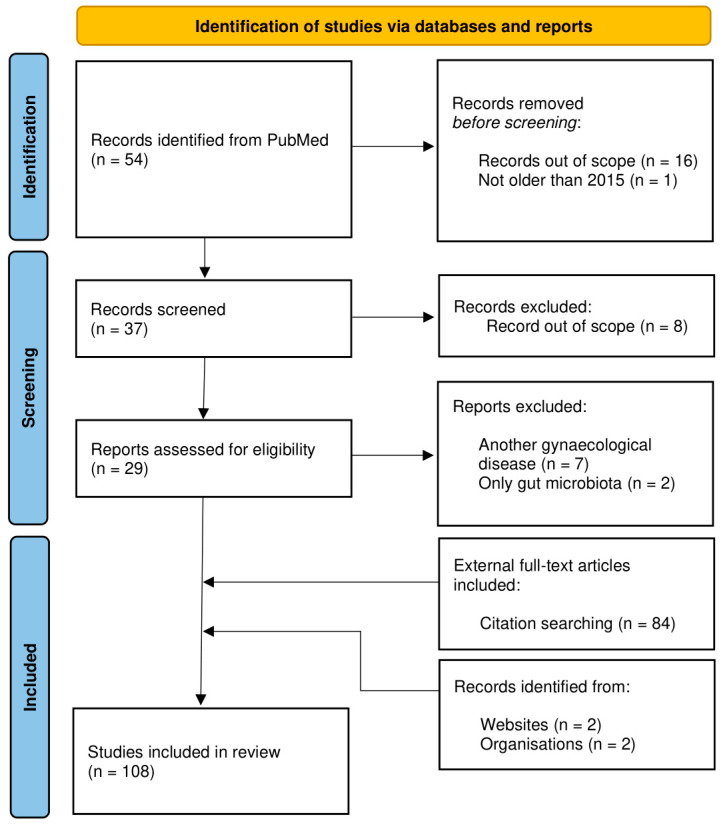
PRISMA flow chart illustrating the study selection process on endometrial cancer and microbiota [12,13]. The study exclusion criteria are included.

The analysis allowing for the exploration of this topic was carried out based on information gathered from the PubMed database. The initial identification of the articles was based on a keyword search for “endometrial cancer” and “microbiota”. Articles published before 2015 were excluded in the first place. Additionally, articles were filtered according to the relevance of the article’s question and discarded as a result. Records focused on other gynecological diseases or only on gut microbiota were excluded from screening. Citation searching provided 84 additional articles, mainly experimental records with immunological input and microbiome composition data. Records from websites with statistical and epidemiological information were also included. As a result, 108 articles were included in this review (Figure 1).

## 3. Endometrial Cancer Classification

The latest morphological and clinical studies have shown that both the traditional histological classification and the two pathogenic subtypes of endometrial cancer do not allow for a reliable assessment of prognosis and response to treatment. Today, histologic classification is predicated on tumor morphology and tumor grade. However, the histopathological report should specify the histological type for endometroid carcinoma: low grade (G1/G2) or high grade (G3) [14]. Tumor grade is an important prognostic feature, and grade 3 tumors are highly aggressive, accounting for a large proportion of endometrial-cancer-related deaths. The molecular classification introduced in 2013 as “The Cancer Genome Atlas” (TCGA) Research Network identifies four molecular categories differing in mutation profile, immunogenicity, and prognosis of EC: POLE ultramutated (POLEmut), mismatch repair deficient (MMRd/MSI-H), P53 mutated (CNhigh), and no specific molecular profile NSMP (CNlow) [15]. These TCGA subgroups and associated molecular alterations correlate with a histologic subgroup of endometrial carcinomas. POLEmut accounts for about 7% of endometrial cancers, usually occurs in young women, and is not strictly related to obesity. These are mainly G3 endometrial tumors with deep myometrial invasion. Serous, clear cell, and mixed carcinomas account for approximately 25% of cancers with POLEmut. Although the histopathological characteristics would indicate a poor prognosis, these cancers have the best prognosis. This is most likely related to the intensification of the cellular immune response in this group of patients. MMRd/MSI-H accounts for 28% of endometrial cancers and is particularly sensitive to immunotherapy when the disease is recurrent or initially advanced. These are mainly endometrioid tumors G1, G2, and G3. In this group, because of the deficit in DNA repair mechanisms, numerous new tumor antigens are formed and expressed, causing a strong immune response. The P53-mutated variant (CNhigh) accounts for 26% of all endometrial cancers. These include cancers with the worst prognosis traditionally—serous carcinomas, mixed histology, and G3 endometrioid carcinomas, and approximately 4% of G1/G2 endometrioid carcinomas. NSMP (CNlow) accounts for 39% of endometrial cancers with an average prognosis. These are mainly G1 and G2 endometrioid carcinomas with frequent CTNNB1 gene mutation, without microsatellite instability [14]. These molecular subgroups provide a gateway for using molecular classification in combination with histopathologic features in routine pathology practice and enhance early diagnostic approaches [15]. Further investigation of molecular analyses for tailored adjuvant treatment strategies may be insightful as well. For example, the POLEmut and MSI-H groups are likely to respond to immune checkpoint inhibitors, as described by Violante Di Donato et al. [16]. Genomic profiling was suggested as a therapeutic guide not only in advanced and metastatic stages but also in precancerous EC lesions involving premenopausal women, who are nulliparous or have pregnancy plans, who would prefer conservative treatment [17].

## 4. Factors Affecting Endometrial Carcinogenesis

### 4.1. Factors Increasing Endometrial Cancer Risk

The American Institute of Cancer Research (supported by the World Cancer Research Fund and the Continuous Update Project), based on 159 EC articles, has drawn conclusions about risk factors and evidence strength [18]. The most convincing evidence was a correlation with body fatness, waist-to-hip ratio, and changes in the level of circulating estrogens as a cause of EC [19]. Obesity influences the level of several hormones and growth factors [20]. Leptin and insulin are raised in these patients, which can promote carcinogenesis. Behind this, insulin resistance is increased in obese people due to abdominal fatness, causing an increase in insulin production in the pancreas (hyperinsulinemia occurs) [21]. The adipose tissue is also the main site of estrogen synthesis in postmenopausal women due to aromatase activity in subcutaneous fat, which increases the conversion of androgen to estrogen [21,22]. It is strongly associated with the risk of EC [23]. Obesity is a low-grade chronic inflammatory state with constant macrophage infiltration into the adipocytes, maintaining anomalous inflammation [24]. The affected cells produce pro-inflammatory factors, such as TNF-α, IL6, C-reactive protein, and leptin [25]. The evidence that greater body fatness, including abdominal fatness and weight gain in adulthood, is a cause of EC is convincing [18]. Another factor (probably a cause of EC) is glycemic and carbohydrate load. Long-term consumption of a high-glycemic-load diet results in hyperinsulinemia, which in turn increases the bioavailability of insulin-like growth factor 1 (IGF-1) and directly promotes cell growth, reduces cell death, and stimulates cell division in EC cell lines [26,27]. Insulin and IGF-1 are powerful negative regulators of sex-hormone-binding globulin synthesis in vitro and may, therefore, stimulate EC [26]. The glycemic load may increase oxidative stress, which is an additional way to promote carcinogenesis [26]. 

Moreover, the American Institute of Cancer Research analysts notify adults’ attained height as a probable cause of EC. The pathogenesis mechanism is caused by developmental factors leading to a greater growth in length in childhood. Taller people have undergone more cell divisions stimulated by IGF-1 and pituitary-derived growth hormone [28], exposing them to more potential for error during DNA replication, leading to cancer development. The number of cell divisions in childhood, nutritional status, health status, and age at sexual maturity can alter the endocrine environment, affecting circulating levels of insulin, estrogens, and growth factors [28]. Recent studies have proven that cancer cells present various resistance mechanisms in anticancer therapies. Reduced oxygen availability may regulate the tumor microenvironment and lead to a more aggressive and metastatic phenotype [29].

### 4.2. Factors Decreasing Endometrial Cancer Risk

On the other hand, the American Institute of Cancer Research did not define any convincing factors decreasing the risk of EC. Nevertheless, they selected two elements with strong evidence for probable protection against EC action—physical activity and coffee consumption. The first one, physical activity included occupational physical activity, walking, and biking [18]. Sustained moderate exercises raise the metabolic rate and increase maximal oxygen uptake [30], which conducts the body’s metabolic efficiency and capacity (possible work perform) outcome. It reduces circulating insulin levels and insulin resistance [31]. Physically active people have increased sex-hormone-binding globulin (the binding protein for estradiol) and lower serum levels of estradiol as a result, but this effect could be mediated by the prevention of weight gain [32]. 

The second factor, coffee consumption was pointed out as a dose–response relationship [18,33]. Coffee consists of several bioactive components, including chlorogenic acid with a strong antioxidant property. It could prevent oxidative DNA damage, inhibit glucose absorption in the intestine, and improve insulin sensitivity [34]. Coffee drinkers have a higher level of sex-hormone-binding globulin, which decreases estradiol exposure and, therefore, reduces EC risk [34,35]. Additionally, coffee consumption is associated with higher levels of adiponectin and lower circulating levels of C-peptide [34]. However, the subject needs further investigation concerning caffeinated and decaffeinated coffee. Including predictable biological mechanisms in the analysis, caffeine is predicted to provide an added reduction in the risk of endometrial cancer [33].

## 5. Uterine Microbiome

An adult human is inhabited by up to 100 trillion microorganisms, including bacteria, viruses, fungi, yeasts, and phages, which are present in the mouth, lungs, digestive tract, genitourinary organs, and on the skin [36,37,38]. EC accounts for almost under 1% of all cancer deaths and 2% of cancer deaths in women [2]. Contemporary reports focus on the role played by the microbiome of patients in the development of EC [36].

Till the second half of the 20th century, before the sterile womb paradigm was challenged, it was thought that the uterus is a sterile niche [39]. However, recent data showed distinct microbial communities’ presence in the female upper reproductive tract (endometrium, fallopian tubes, ovaries) [40]. It was observed that the cervical plug inhibits but does not block the access of vaginal bacteria to the uterine body; therefore, the uterus is not sterile [41,42]. This seems to be proved by the seminal microbiome presence in the uterus, supported by peristaltic moves in the cervix [43,44,45]. Besides spreading with sperm and through the cervix, bacteria probably come into the endometrium from the intestine or oral cavity transported via the bloodstream or through retrograde transmission through the fallopian tubes, insertion of an intrauterine device, or gynecological procedures (e.g., assisted reproductive technology) [8,46,47,48,49,50]. 

The microbiome of the female reproductive tract is reported to be site specific. Even though species of bacteria are in a continuum between the upper and lower reproductive tract, there are significant differences between the diversity and proportion of these species [51]. However, there are ongoing discrepancies between experimental studies about the statement of continuum or independent microbiome communities within the vagina–uterus tract. The issue remains unsolved and demands more investigations for a conclusion. However, compared with the cervical and vaginal microbiota, a wider diversity and complexity of bacteria is reported in the endometrium [40,51]. The environmental condition may also be connected, such as the endometrium’s pH value, temperature, humidity, and abundant blood flow [52]. Moreover, periodic changes in its components are suspected, resulting from changing hormonal exposure or pregnancy [53]. During the menstrual cycle, the endometrial tissue undergoes dynamic changes: rapid proliferation, secretory transformation, angiogenesis, interstitial edema, or desquamation. This affects the composition not only of the microbiota but also of immunocompetent cells and the expression of inflammatory genes [54,55].

### 5.1. Microbiome of a Healthy Uterus

The vaginal microbiome of reproductive-age, healthy women is dominated by the *Lactobacillus* genus [56]. Both vaginal and cervical mucus samples represent *Lactobacillus* over 99.9%, which is a low α-diversity sign. Mostly *L. crispatus* (39.86%) and *L. iners* (29.85%) were reported within the genus [40]. Cervical mucus drawn from the cervical canal showed less *Lactobacillus* domination generally and more diversity within individuals. This tendency toward diversity continues into the endometrium; however, the overall quantity of bacteria is ~10,000-fold lower than the estimated number of organisms in the vagina [40]. That is why the uterus is considered a low-abundance site that, untruthfully, seems sterile [39,57,58,59,60,61,62,63,64,65,66,67]. Nowadays, uterine microbiome investigation is undertaken with high-quality detection methods, such as 16S ribosomal RNA gene sequencing or fluorescence in situ hybridization with 16S rRNA-targeted probes. However, the healthy women’s upper reproductive tract (endometrium, fallopian tubes, and ovaries) microbiome is not fully characterized yet [51]. Recently published data point to a higher microbial diversity in the upper reproductive tract than in the lower one (vagina and cervix) [40]. However, the suggested composition differs between studies, and it remains unclear whether some species are transient colonizers or genuine members of the uterus. Commonly reported in the uterine samples was *Lactobacillus,* specifically for the vagina and cervix, but in a much smaller part relatively (30.6% of total), and there was a downward trend continuum in the fallopian tubes (1.69% of total) [40].

So far, mainly bacteria of the *Lactobacillus* sp. genus have been identified in the endometrium, but their number has been significantly reduced compared to the population in the vagina (30.6%: 99.97%) [36,40]. Chen Chen et al. conducted an analysis of 110 women of reproductive age, detecting microbiome composition, nature of colonization, and cultivation of the microbiome in the women’s reproductive tract [40]. In the uterus, they identified a notable fraction of *Pseudomonas* (9.09% of the total), *Acinetobacter* (9.07% of the total), *Vagococcus* (7.29% of the total), and *Sphingobium* (5% of the total). Among the rest of the bacteria, the noticed species were *Comamonadaceae*, *Arthrobacter*, *Dysgonomonas*, *Shewanella*, *Pseudomonadaceae*, *Delftia*, *Tissierellaceae*, *Sphingomonas*, *Erysipelotrichaceae*, and *Erysipelothrix* (collectively 38.95% of the total) [40].

### 5.2. Microbiome of a Pathological Uterus

The uterine microbiome’s composition remains unclear but seems to vary in different pathologies [8]. The latest data of Walther-António MRS et al. reported several bacterial taxa correlated with EC. They mentioned the taxa *Firmicutes* (*Anaerostipes*, *Dialister*, *Peptoniphilus*, *Ruminococcus*, and *Anaerotruncus*), *Spirochaetes* (*Treponema*), *Actinobacteria* (*Atopobium*), *Bacteroidetes* (*Bacteroides and Porphyromonas*), and *Proteobacteria* (*Arthrospira*) [68]. Besides those, the co-culture of *Atopobium vaginae* and *Porphyromonas* sp. (99% match to *P. somerae*) was significantly associated with EC, especially in a high vaginal pH (>4.5) [68]. However, their research was based on samples obtained from the vagina, cervix, fallopian tubes, and ovaries. 

**Figure 2:**
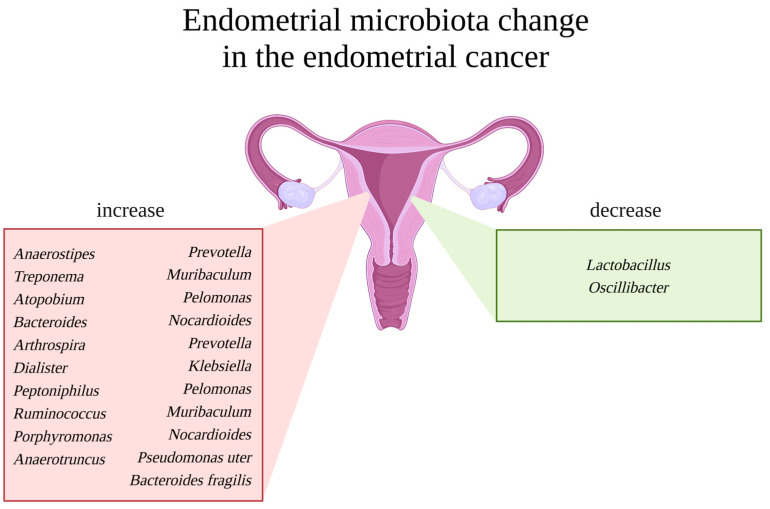
Endometrial microbiota change in the case of endometrial cancer. Uterus in the middle. On the left, a red box with a list of representative bacterial genera reported to be increased in endometrial cancer patients. On the right, a green box with a list of representative bacterial genera reported to be decreased in endometrial cancer patients. Created with BioRender.com.

Another study, by Mitchell et al., identified more bacteria (besides *Lactobacillus* sp.) of the genera *Gardnerella* sp., *Bifidobacterium* sp., *Streptococcus* sp., and *Prevotella* sp., but the pilot data are not clear and consistent as to their effect on cancer formation, fertility, maintenance of pregnancy, or stimulation of the immune system [36,69,70]. According to Wanting Lu et al., *Micrococcus* sp. was identified as specific to EC patients. Higher mRNA levels of the pro-inflammatory and oncogenic cytokines interleukin-6 (IL6) and interleukin-17 (IL17) have been shown to be associated with these bacteria [71]. The abovementioned and additional studies are summarized in Figure 2. 

Overall, the available studies’ results differ, and uterine bacteria demand further investigation. The commonalities in any endometrial disease are only a decrease in the amount of *Lactobacillus* or *Firmicutes* and an increase in *Proteobacteria* (*Staphylococcus*, *E. coli*, etc.), *Bacteroidetes* (*Bacteroides fragilis*, *Prevotella*, *Bacteroides*, etc.), and *Actinobacteria* (*Garnerella*, *Bifidobacteria*, etc.) [8]. Undoubtedly, the microbiome plays a role in the physiology of the endometrial epithelium, suggesting its importance in uterine pathogenesis. It is promising in disease detection in the upper reproductive tract. How exactly does the microbiome act in this specific and dynamic niche?

## 6. Implications of Microbiome

It is crucial to perceive bacteria in the environment while considering their impact. Individual bacteria exist in colonies and guilds, co-existing with other species and microorganisms (allies and opponents), surrounded by dynamically changeable chemical compounds and proliferating tissues, varying, in particular, in pH, humidity, and temperature, in a limited space with limited sources. Moreover, the current view is an established result of evolution and our co-existence with bacteria [72]. Bacteria in population density can proceed into quorum sensing and share the regulation of gene expression on a wider scale [73]. We harbor symbionts, neutrals, and pathogens. The explanation of the microbiome’s activity in the uterus should be supported by microenvironment awareness in parallel. Furthermore, it should consider changes in condition, constant stimulation, and response. The microbiome is a community in which both producers and recipients are involved, active, and concerned.

The endometrium consists of two types of cell layers: basal and functional, surrounded by the myometrium and the serosa. One-third of the endometrium is basal, near the myometrium, and the rest is the spongy, dense part [8,74]. The thickness of the endometrium changes during the menstrual cycle due to the dynamics of the hormonal levels. The cycle consists of proliferation, differentiation, and shedding (menstruation) [74,75]. The first of the microenvironment compounds are hormones. The endometrial epidermis, blood vessels, stroma, and glands undergo proliferative changes provoked by estradiol in the follicular phase [8]. The combination of the FSH and LH concentration peaks triggers ovulation. Afterward, the higher concentration of progesterone starts the luteal phase while the endometrium continues to thicken, the stroma becomes more edematous, the glands grow and bend, the spiral arterioles further grow and curl, and the vascular lumen expands [8,76]. It is a specific period during which the uterus adjusts for the implantation of the blastocyst, followed by fetal growth and survival [8]. If implantation does not occur, hormone levels drop, and the endometrium exfoliates. During the secretory period, the functional layer disintegrates and falls off from the basal layer. Ischemia necrosis and denudation of the distal vascular wall and tissue occur, forming menstruation [8]. The bacteria in the endometrium participate in the apoptosis and proliferation of the endometrial cells during the menstruation cycle [6,7]. However, the mechanism behind this is not defined yet. Moreover, the microbiota engages in embryo implantation and pregnancy maintenance [77,78]. A high abundance of *Lactobacillus* in the endometrium is associated with better reproductive outcomes, whereas microbiota imbalance portends pathological events [52,69,79].

The immunological system plays a key role in endometrium physiology. Immune cells are scattered within the endometrium [8]. They are composed mainly of innate immune cells such as neutrophils (NEUs), dendritic cells (DCs), macrophages (Ms), mast cells, uterine natural killer cells (uNKs), and adaptive cells: T cells and B cells. Their functions are physiological immune microenvironment maintenance, endometrial remodeling, decidualization, embryo implantation, regulation of the invasion of the trophoblast, enhancing vascular remodeling through the extravillous trophoblast, protecting against infection, and promoting maternal–fetal immune tolerance [8,80]. At the same time and in the same space, bacteria live in cohorts, supplying common communications, and sharing the surface. Favorable species occupy the endometrial area and prevent pathogenic microorganisms from attaching, providing anti-infection abilities [6,7,8]. On the other hand, microbial ligands can be bound to host receptors and take part in an immune response by triggering the production of chemokines, inflammatory cytokines, and antibacterial substances [6,7]. Together, the immune system and the microbiome cooperate in two parallel goals: elimination of pathogenic microorganisms and promotion of immune tolerance of the semi-allogenic growing fetus [8,80]. The interaction between the immune cells and the endometrial microbiota is based mainly on toll-like receptors (TLRs), the complement system, antimicrobial peptides (AMPs), bacterial DNA, proteins, and lipopolysaccharides (LPSs) [81].

## 7. How Does the Endometrial Tissue Become Malignant?

**Figure 3:**
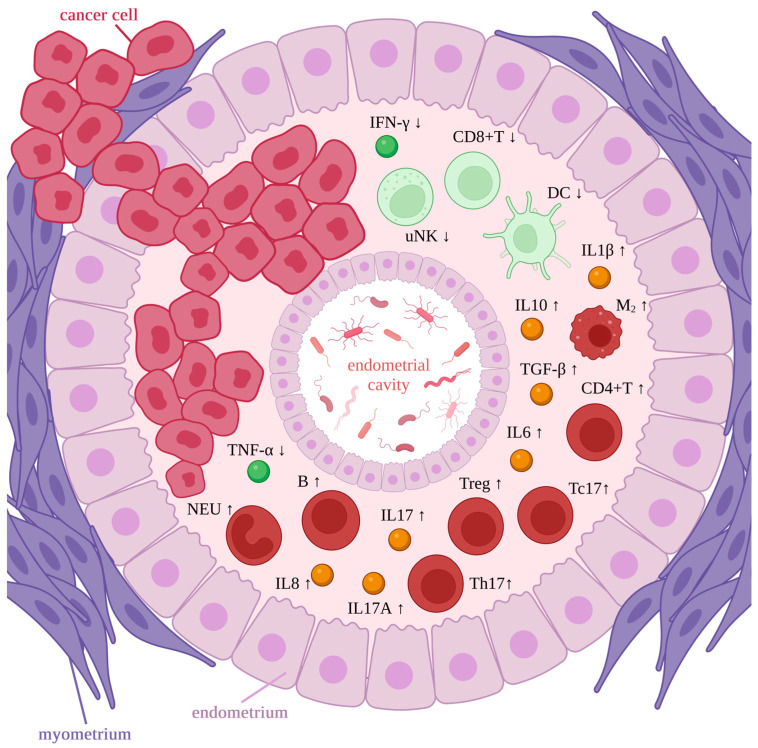
Immune response in endometrial cancer. Microbiota representation in the endometrial cavity. Rings of endometrial tissue surrounded by the myometrium ring. Cancer cells within the tissues. Immune cells and inflammatory factors within the endometrium. High concentration is marked as red/yellow and bold, while low concentration is marked as green and pale. Tc17—CD8-positive T cell producing IL17, IFN-γ—interferon-γ, IL6—interleukin-6, uNK—uterine natural killer cell, CD8+T—CD8-positive T cell, DC—dendritic cell, IL10—interleukin-10, IL1β—interleukin-1β, TGF-β—transforming growth factor-β, M2—phenotype 2 macrophage (mainly tumor associated macrophages), CD4+T—CD4-positive T helper cells, Treg—regulatory T cells, Th17—T helper cells producing IL17, IL17—interleukin-17, IL17A—interleukin-17A, IL8—interleukin-8, B—B cell, NEU—neutrophil, TNF-α—tumor necrosis factor-α. Created with BioRender.com.

The microbiome and EC development are related through local microbial imbalance and immune inducement. The microbiota, which stimulates inflammation, may induce immunopathological changes, which lead to tumors eventually [9]. It is modulated through inflammatory factors, such as increased IL6, IL17, interleukin-17A (IL17A), interleukin-10 (IL10), interleukin-8 (IL8), transforming growth factor-β (TGF-β), and interleukin-1β (IL1β) and decreased interferon-γ (IFN-γ) and tumor necrosis factor-α (TNF-α) [8]. The immune cells respond by increasing phenotype 2 macrophages, mainly tumor-associated macrophages (M_2_s), regulatory T cells (Tregs), CD4-positive T helper cells (CD4+Ts), B cells (Bs), neutrophils (NEUs), T helper cells producing IL17 (Th17s), CD8-positive T cells producing IL17 (Tc17s), and decreased CD8-positive T cells (CD8+Ts), uterine natural killers (uNKs), and dendritic cells (DCs) [8] (Figure 3). Tumor-associated macrophages can promote carcinogenesis and inhibit the cytolytic T-cell response. The growing tumor is maintained through the frequent infiltration of lymphocytes within cells and the peritumoral area [82,83,84]. Apart from the abovementioned roles, uNKs, DCs, M_2_s, NEUs, T cells, and B cells play a key role in the immune response with the companionship of pro-inflammatory (interleukin-1, IL6, IFN-γ) and anti-inflammatory cytokines, which vary widely in concentration levels [8]. If inflammation occurs, malignant transformation is standard, but the pathways specific to the endometrium require further investigation. 

Ben et al. presented a study about human leukocyte antigen G. It is an immunosuppressive molecule, with reported higher concentrations in EC patients than in healthy people, and it differs according to the cancer stage [85,86]. This molecule could be involved in the early invasiveness of cancer. Hopefully, the studies focused on its role will be continued. Other examples are ErbB receptors classified as a subclass of receptor tyrosine kinases and components of the EGF system signaling network in cells. The expression of ErbB receptors was reported to be significantly different in EC, compared with that in the premenopausal and postmenopausal endometrium [87,88,89]. ErbB-2 overexpression and ErbB-2 gene amplification were reported extensively within EC patients as indicators of a more aggressive disease with reduced response to treatment and less favorable outcomes (especially in patients with type II EC in the traditional classification) [87,90,91,92]. The linkage between microbiome transcriptional activity and ErbB gene expression is worth further investigation due to the role of the EGF system in the regulation of endometrial cyclical growth and its associations with the widely described PIK3CA-PIK3R1-PTEN, RTK/RAS/ β-catenin signaling pathways. Finally, analysis of the interdependence between the microbiome and the immune system, including gene expression changes and signaling pathways, is needed in this field.

## 8. How Does the Gut Influence Endometrial Tissue Carcinogenesis?

Bacteria colonize both the intestine and the colon. Changes in the composition of the intestinal microbiota also reduce the amount of suppressor substances in the intestines, like short-chain fatty acids (SCFAs) [93]. Overall, 90–95% of SCFAs in the colon are propionates, acetates, and butyrates. Bacteria produce them by fermenting nondigestible carbohydrates, proteins, and peptides [93,94,95]. Their function is to maintain a low pH in the gut, which allows the growth of bacterial phyla promoting homeostasis, including *Lactobacilli* and *Bifidobacteria* [93,96,97]. On the other hand, SCFAs hinder colonization by opportunistic pathogenic types, such as *Clostridium* or *Escherichia coli* [94]. Moreover, SCFAs are involved in the preservation of a functional gut barrier. They stimulate the regeneration of epithelial cells and the production of mucus and antimicrobial peptides [93,94,98]. On a systemic scale, these protective effects inhibit the translocation of toxins and bacteria into the bloodstream and, as a result, prevent cancer, obesity, chronic inflammation, and metabolic syndrome [94,98,99,100]. 

Gut dysbiosis occurs when an imbalance in the microbial community becomes persistent, the stability and diversity of colonies decrease, and opportunistic pathogenic bacteria obtain the potential for overgrowth [101,102,103]. It was observed in several studies that pathogenic bacteria reduce SCFA production and bile acid concentration in the intestinal lumen [93,104]. An altered gut microbiome enhances the inflammatory response through microorganism-associated molecular patterns. These are activated by pattern recognition receptors, such as toll-like receptor 4 (TLR-4) and its ligand, LPS. The following pathway initiates the inflammatory response and leads to the upregulation of pro-inflammatory cytokines (including IL17, TNF-α, and IFN-γ) [105]. These potentially alter the vaginal microbiota by increasing the production of ovarian steroid hormones in the ovaries [93]. TNF-α and IL6 synergistically promote the expression of aromatase, 17β-hydroxysteroid dehydrogenase, and estrone sulfatase (enzymes involved in ovarian steroidogenesis) [106]. This creates a stimulation loop. Behind this phenomenon, elevated estrogen levels can support the development of endometrial carcinoma indirectly. It can influence the endometrium through the gut–vaginal microbiome axis [10,11], which was shown to present different microbial compositions within healthy individuals and patients with cancer. 

The intestinal microbiota also plays a key role in the level of circulating estrogen through the secretion of β-glucuronidases [11]. This enzyme deconjugates estrogen, activating it and allowing it to attach to its receptors. Disturbed composition of the intestinal microbiota and lower diversity adversely affect the level of circulating estrogen, which could be considering as a contribution to the development of obesity, metabolic syndromes, cognitive dysfunction, fertility problems, polycystic ovary syndrome, and the development of cancer [11]. A preclinical study by Casaburi et al. explored the role of some other bacterial metabolomic products in cancer development [107]. A low dose of chenodeoxycholic acid activating the TGR5/GBPAR1 pathway enhanced cell proliferation in Ishikawa EC, while a high dose provided a cytotoxic effect [93,107]. Another product, butyrate, has shown an antitumoral effect. It inhibited histone deacetylase and contributed to moving tumor cells from the S-phase to the G0/G1 and/or G2/M phases [108]. Moreover, another study provided data about ornithine decarboxylase in EC. This enzyme is mostly derived from *Shigella flexneri*, *Shigella sonnei*, *Escherichia coli*, and *Streptococci*. Ornithine decarboxylase is involved in putrescine biosynthesis and related to *MYC* gene amplification, both contributing to a preneoplastic effect [93,109]. The gut microbiome should be considered to be interconnected with the upper genital tract. The metabolic, immunological, and hormonal perturbations of the intestinal microbiome may contribute to carcinogenesis in the reproductive system [93,110].

## 9. Limitation of Current Evidence

There is a lack of significant experimental results in research on uterine microbiota and EC. Due to the uterus’s anatomical site, being the upper reproductive tract, sample collection is difficult. Moreover, there is no unified standard for the detection and sampling methods. The proximity of the vaginal and cervical niche makes it more difficult and less trustworthy. Wrong sample collection could lead to unconsciously falsified results [111,112]. Samples are susceptible to DNA contamination from the background of the collection and laboratory procedures. The technical method for identifying endometrial microbiota is not widely used, and as a result, the number of studies is low. Accordingly, the sample size is usually insufficient. The interpretation of the obtained data remains challenging too. The complex mechanisms of the endometrium, microbiota, and immune system’s co-existence need further research. Moreover, individual microecology varies and posing another obstacle to the reliability of the conclusions. A compendium of future perspectives on the mentioned limitations is widely proposed by Zhu et al. in the article titled “Iron triangle” of regulating the uterine microecology: Endometrial microbiota, immunity and endometrium [8].

## 10. Conclusions

Our understanding of the complex relationship between different host-intrinsic microorganisms, as well as the multifaceted mechanisms by which they influence health and disease, has grown tremendously, hastening the development of novel personalized therapeutic approaches in cancer treatment. Accordingly, the evaluation of a patient’s microbial composition and function and its subsequent targeted modulation represent key elements of future multidisciplinary and personalized-medicine approaches.

It was proved that malignancy of the endometrium as a hormone-dependent tissue is correlated with obesity, metabolic dysregulation, and constant inflammation processes. Discovery of the uterine microbiome enriches data about bacterial contributions to EC. The bacterial composition varies in different pathologies, a topic that remains under further investigation. The newest discoveries provide data about species’ presence in the gut, vagina, and uterus. By analyzing metabolic pathways and the behavior of the microbiome, we can presume their physiological impact. Both the intestinal and the gynecological microbiomes could be potentially influential in carcinogenesis through estrogen metabolism regulation, inflammation incitement locally, or accompanying disease conduction. The uterine microbiome is under further investigation, providing more data about the bacteria composition and influential pathways. The combination of those may lead to the development of EC prognostic and therapeutic agents. 

## 11. Future Directions

Many aspects remain unresolved. What are the patterns of microbiome compositions in particular gynecological diseases? What is the primary cancer inflammatory process or prooncogenic bacterial change? How does the correlation between them develop, and when does it start? Is obesity a trigger for hormonal changes or is obesity the result of inflammation caused by bacteria? How do the gut and uterine microbiome populations coexist, and are there any transfers or communication between bacteria in the bloodstream? The uterine microbiome’s correlation with EC progression demands further investigation in the field. Nevertheless, if the microbiome is to be successfully translated into next-generation oncologic treatments, a new multimodal model of the oncomicrobiome must be conceptualized that incorporates the cometabolism of pharmacologic agents into cancer care. 

## Data Availability

No new data were created or analyzed in this study. Data sharing is not applicable to this article.
